# Perforated Concave Earplug (pCEP): A Proof-of-Concept Earplug to Improve Sound Localization without Compromising Noise Attenuation

**DOI:** 10.3390/s23177410

**Published:** 2023-08-25

**Authors:** Nir Fink, Rachel Levitas, Arik Eisenkraft, Linn Wagnert-Avraham, S. David Gertz, Leah Fostick

**Affiliations:** 1 Department of Communication Disorders, Acoustics and Noise Research Lab in the Name of Laurent Levy, Ariel University, Ariel 40700, Israel; 2 Israel Defense Forces Medical Corps, Hakirya 6473424, Israel; rachelevitas@gmail.com; 3 Institute for Research in Military Medicine (IRMM), Faculty of Medicine of The Hebrew University of Jerusalem and the Israel Defense Forces Medical Corps, Jerusalem 9112102, Israel; arik.eisenkraft@mail.huji.ac.il (A.E.); linn.wagnert@mail.huji.ac.il (L.W.-A.); davidg@ekmd.huji.ac.il (S.D.G.); 4 The Saul and Joyce Brandman Hub for Cardiovascular Research and the Department of Medical Neurobiology, Institute for Medical Research (IMRIC), Faculty of Medicine, The Hebrew University of Jerusalem, Jerusalem 9112102, Israel; 5 Department of Communication Disorders, Auditory Perception Lab in the Name of Laurent Levy, Ariel University, Ariel 40700, Israel; leah.fostick@ariel.ac.il

**Keywords:** hearing protection devices, sound localization, military noise, perforated concave earplugs (pCEP)

## Abstract

Combat soldiers are currently faced with using a hearing-protection device (HPD) at the cost of adequately detecting critical signals impacting mission success. The current study tested the performance of the Perforated-Concave-Earplug (pCEP), a proof-of-concept passive HPD consisting of a concave bowl-like rigid structure attached to a commercial roll-down earplug, designed to improve sound localization with minimal compromising of noise attenuation. Primarily intended for combat/military training settings, our aim was an evaluation of localization of relevant sound sources (single/multiple gunfire, continuous noise, spoken word) compared to 3M™-Combat-Arms™4.1 earplugs in open-mode and 3M™-E-A-R™-Classic™ earplugs. Ninety normal-hearing participants, aged 20–35 years, were asked to localize stimuli delivered from monitors evenly distributed around them in no-HPD and with-HPD conditions. The results showed (1) localization abilities worsened using HPDs; (2) the spoken word was localized less accurately than other stimuli; (3) mean root mean square errors (RMSEs) were largest for stimuli emanating from rear monitors; and (4) localization abilities corresponded to HPD attenuation levels (largest attenuation and mean RMSE: 3M™-E-A-R™-Classic™; smallest attenuation and mean RMSE: 3M™-Combat-Arms™4.1; pCEP was mid-range on both). These findings suggest that the pCEP may benefit in military settings by providing improved sound localization relative to 3M™ E-A-R™-Classic™ and higher attenuation relative to 3M™-Combat Arms™-4.1, recommending its use in noisy environments.

## 1. Introduction

Military service members are exposed to a broad range of complex signals, some of which are signals of interest and some are merely unwanted noise. Common signals of interest that are vital for mission success include spoken commands, radio communication, and the exchange of tactical information related to missions. Such signals need to be heard clearly; hence, hearing acuity is a critical component of combat effectiveness and mission success [1,2]. In this context, Brungart et al. [3] recently developed an evidence-based military auditory fitness-for-duty standard based on the 80-word modified rhyme test (MRT). This criterion was based on minimum speech intelligibility requirements that have been established for military communications equipment used by listeners with normal hearing. On the other hand, unwanted signals (i.e., noise) interfere with signals of interest and need to be attenuated. Steady-state noise levels should be reduced to less than 85, A-weighted decibels (dBA), and peak-pressure levels of impulsive noise should be less than 140 dBP at the ear (protected or unprotected) [4]. Attenuating military noise is a challenging task since such noise is of multiple types, sometimes heard concurrently, such as impulse noise from operating weapons and explosions of munitions, or continuous noise from armored vehicle engines [4,5]. In this context, a soldier may be viewed as an “acoustical sensor”, providing useful or even critical information, because acoustic information sensed by each soldier, especially when spaced apart, serves as essential input for commanders in localizing sounds and understanding the surroundings.

However, exposure to excessive military noise is dangerous and may result in acute acoustic trauma, permanent hearing loss, tinnitus, hyperacusis, recruitment, distortion, or abnormal pitch perception [1,6,7,8,9,10,11]. As isolating oneself from noise or extending the distance between oneself and a noise source is often unfeasible in military scenarios, noise is usually attenuated by using a hearing protection device (HPD) such as an earplug or earmuff, or through the use of double HPDs (earplug and earmuff). When commercial HPDs are not present, makeshift HPD solutions, such as inserting fingers into the ear canals or pressing the finger against the tragi, are improvised [12,13]. The degree of attenuation provided by HPDs varies between a few dBs and up to 30 or even 40 dB at specific frequencies.

Improper insertion of HPD may result in poor attenuation of military noise, leading to both positive and negative consequences: improved sound localization on one hand (i.e., better localization of less attenuated signals from the surroundings) and possible hearing damage on the other hand. Proper use of HPDs, as instructed by the manufacturer, protects the service member from auditory damage but can also cause missed verbal cues and warning signals, the latter of which may be a reason for intentional incorrect/partial insertion—or even temporary removal—of the HPD. Service members will often use HPDs only if their use sufficiently preserves their ability to communicate and detect sounds, and if they are compatible with other gear [1,14,15,16]. The use of HPDs raises a constant dilemma: Does this HPD present sufficient noise attenuation? Does it enable sufficient speech intelligibility and sound localization? Reports show that issuing an excellent HPD in terms of noise attenuation resulted in cases where combatants and civilian personnel removed the HPD for better sound localization and speech intelligibility, which resulted in hearing damage [10]. Therefore, an HPD that excels in attenuation may, in fact, indirectly cause hearing damage since the users need to remove it for additional auditory information. It should be noted that reduced localization abilities can also be caused by non-HPD-related sources such as hearing loss, consisting of a temporary threshold shift (TTS) or even a permanent threshold shift (PTS), which often manifests as limited bandwidth access, namely, an inability to detect high-frequencies [17].

HPDs can be subdivided into noise-level-dependent and noise-level-independent attenuation devices [18,19], with both kinds of passive HPDs acting as physical barriers that aim to prevent the propagation of noise into the ear canal. With noise-level-independent HPDs, constant energy reduction is achieved for different SPLs [20]. In contrast, noise-level-dependent HPDs contain an orifice with a narrow channel throughout the length of the earplug that provides less attenuation of low frequencies, thus serving as a low-pass filter [21], and less attenuation of lower intensity SPLs, e.g., speech or low SPL environmental noise. Zimpfer and Sarafian [19] cite Dancer and Hamery [22] who claim that the acoustic impedance of the cavity (narrow channel) is related to its viscous resistance, which has a non-linear component proportional to the particle velocity. Such level-dependent HPDs are often called non-linear earplugs because of their ability to reduce high-intensity weapon noise. Passive level-dependent earplugs allow for better hearing of speech and environmental sounds, yet protect from sudden impulse noise from small- to large-caliber weapons, a reason for their popularity in the military [4]. At low SPLs, orifice and channel dimensions allow sound waves to move through the earplug without interruption, but when the SPL equals or exceeds 110 dB, the passive level-dependent mechanism kicks in as the pressure acts on the plug, causing the air at the plug’s entrance to move circularly. This prevents the passage of sound waves and provides amplitude sensitivity [18,22,23,24].

In light of these limitations and of extended military incursions resulting in large numbers of service members presenting with hearing complaints attributed to acute acoustic trauma from military noise exposure [10,11], we aimed to develop a specially-designed proof-of-concept earplug for addressing these limitations [25]. The primary objective was to improve impulse and continuous noise attenuation compared to commercial earplugs without compromising speech understanding and sound localization. Other objectives, beyond the scope of the current study, included ensuring the stability of the earplug in the ear canal and enabling tactical radio communication to be heard clearly when using ear protection in combat scenarios. Consequently, the perforated concave earplug (pCEP)—not to be confused with the abbreviation CEP used for communication earplugs—was designed by the first author to address these challenges.

### 1.1. pCEP Design

**Figure 1:**
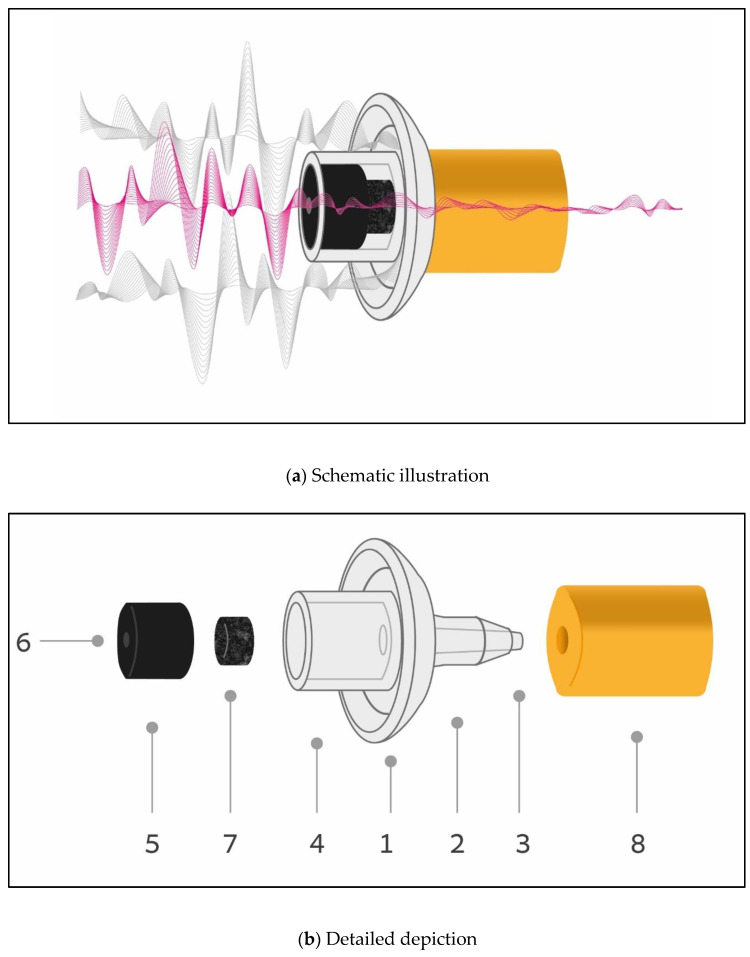
Schematic (**a**) and detailed (**b**) illustrations of the pCEP (details on parts labeled (1–8) are presented in the text).

The pCEP design is depicted in Figure 1a. It is a passive HPD, consisting of two elements: (1) a concave bowl-like rigid structure attached to (2) a commercial roll-down earplug that has been perforated lengthwise. Given proper evaluation of the earplug and its combination with the rigid structure, any commercially available roll-down earplug amenable to perforation is suitable for use. In Figure 1a, loud sound waves (depicted as gray waves) are either reflected by the rigid concave-like bowl structure or are attenuated as they pass along the inner narrow tube of the pCEP (depicted as red waves). Figure 1b depicts a layout and further data on the pCEP’s components. For better visibility of the components, the roll-down earplug has been “removed” from the pCEP to enable full viewing of the concave rigid structure. Moreover, inner parts (5) and (7) within the concave rigid structure have been extracted from part (4). The mid-portion of the rigid structure is a concave-like bowl (1) facing the lateral side. Proper (deep) insertion of the roll-down earplug into the ear canal ensures that the bowl-like structure is situated firmly in the concha and remains pressed against the tragus; this placement is crucial for soldiers undergoing physical stress, as it prevents the earplug from being dislodged from the ear canal by sudden, forceful movement or adjustment of other protective headgear (e.g., helmet). Opposite the bowl-like structure (1) is a narrow tail-like hollow tube (2) attached to the inner portion of the roll-down earplug. Within this hollow tube (2) is placed a narrower tube (3) with an outer diameter of 1.35 mm and an inner diameter of 0.5 mm. This narrower tube is used as a channel to convey soft sounds such as speech. An orifice of a narrower tube (such as 3) was suggested to provide a controlled leakage path that changes sound attenuation behavior [21]. Another hollow tube, serving as a grip handle (4) and wider in its external diameter than the other hollow-tube (2), faces the lateral side of the rigid structure. The grip handle (4) is held by the thumb and index finger and is used to push the roll-down earplug into the ear canal. The center of the bowl-like structure (1) is perforated, thereby enabling a straight passage of soft sounds along the interior length of the earplug. Two additional components are added within the tube (4). The first, a black rubber hollow cylinder (5) with a narrow opening (6), allows for the firm fitting of a commercial “listen only” acoustic tube—an accessory supplied by tactical radio manufacturers for the dismounted soldier. One end of the “listen only” acoustic tube is connected to the narrow opening (6) using a simple passive adaptor (see Figure S1), while the other end is connected to the audio output of a voice tactical radio, for example, a personalized networking radio such as Elbit Systems—Land and C^4^ ™ PNR-1000™. The second component added within tube (4) is a small noise filter (7) used to provide further sound attenuation before passing through narrow tube (3). Such a small noise filter was suggested by Berger and Hamery [23] to change sound attenuation behavior as is the case with the “Institute Saint Louis (ISL) filter” in the 3M™-Combat-Arms™4.1 earplug.

The bowl-like structure serves as the first barrier to unwanted noise reaching the ear by acting as a “reflector of sound” (similar to the technology used to reflect light with a reflector lens), followed by the second barrier of the roll-down earplug. The center of the bowl-like structure is perforated, enabling a straight passage for soft sounds along the interior length of the earplug. The displayed prototype is an open-mode version of the pCEP since the tube is open on both ends.

### 1.2. The Current Study

In this study, we aimed to evaluate the performance of the proof-of-concept, passive perforated concave earplug (pCEP) in a military-related localization task, in the context of human factor component examination of this proof-of-concept prototype design. As the prototype earplug is intended primarily for use in combat and military training, our primary goal was to evaluate the localization of relevant sounds including single and multiple gunfire noise, continuous noise, and spoken word. As the primary purpose of an HPD is protection against the detrimental effects of exposure to loud noise, we first evaluated the attenuation level of the pCEP, as compared to the standard commercial earplugs issued to combatants in the Israeli Defense Forces (IDF): the 3M™-Combat-Arms™4.1 earplugs in open-mode, and the 3M™ E-A-R™ Classic™ earplugs (Experiment 1). We then proceeded to the main goal of the current study: to compare the localization performance of the pCEP compared to these two commercial earplugs (Experiment 2).

We hypothesized that (1) the pCEP would provide less attenuation than the 3M™ E-A-R™ Classic™ earplug and higher attenuation than the 3M™-Combat-Arms™4.1 earplugs in open-mode, and (2) the pCEP would enable improved localization results compared to the 3M™ E-A-R™ Classic™ earplug and worse localization results compared to the 3M™-Combat-Arms™4.1 earplug.

## 2. Experiment 1: Attenuation

The aim of Experiment 1 was to compare the attenuation levels between HPDs. Subjective attenuation levels of the pCEP were measured in the Real Ear Attenuation at Threshold method (REAT) and were compared to published data of 3M™-Combat-Arms™4.1 [26] and 3M™ E-A-R™ Classic™ earplug [27]. This method involves the human perception of the sound threshold with and without HPDs. Objective attenuation levels were obtained for all HPDs with the Artificial Test Fixture (ATF) method. This method uses a Head and Torso Simulator (HATS) (serving as an ATF) to measure sound stimuli at louder sound levels than the threshold of hearing.

### 2.1. Materials and Methods

#### 2.1.1. Participants

REAT measurements were performed on twenty participants (70% female) aged 20–35 years, who were screened for normal hearing (thresholds ≤ 25 dB HL at frequencies of 500, 1000, 2000, and 4000 Hz) and were enrolled in Experiment 1.

#### 2.1.2. Stimuli

REAT measurements for subjective attenuation levels were measured in response to pure tones at frequencies of 125, 250, 500, 1000, 2000, 4000, and 8000 Hz. White noise was used for ATF measurement.

#### 2.1.3. Hearing Protection Devices (HPDs)

Three HPDs were tested in the current study: the proof-of-concept pCEP earplug, the 3M™-Combat-Arms™4.1 earplug in open mode, and the 3M™ E-A-R™ Classic™ earplug.

#### 2.1.4. Apparatus

To test the subjective attenuation level of the HPDs at each frequency (REAT method), a GSI-61 Audiometer was used to produce pure tones in the sound field using an RCF Ayra 5^®^ active monitor (1” tweeter; 5” woofer), positioned 60 cm in front of the participant. Participants’ responses were obtained with the audiometer’s response button. Objective attenuation levels (ATF method) were recorded using a Brüel and Kjær HATS type 4128C connected to a Brüel and Kjær dual microphone supply type 5935 L. The HATS was connected to the SINUS APOLLO™ measuring system, and SINUS SAMURAI™ software was used to analyze the measured signal. The HATS was calibrated with a GRAS 42AA Pistonphone, producing a 114dB signal at 250 Hz. Both subjective and objective attenuation measurements took place in the same sound-attenuating anechoic chamber.

#### 2.1.5. Procedure

The study protocol for the subjective measurement of attenuation (REAT) was approved by the university ethics committee before the commencement of the research. After obtaining signed informed consent, participants’ hearing thresholds (in dB HL), defined as the lowest signal intensity at which the signal can be identified 50% of the time, were measured for each frequency, with and without each HPD. This procedure was carried out twice to verify the thresholds. HPDs were inserted in ear canals by the experimenter to ensure proper HPDs fit across participants. The objective measurement (ATF method) for the pink noise, gunshots, and spoken word were also measured with and without HPD using the HATS. Attenuated SPLs were subtracted from unconcluded SPLs to obtain the attenuation of each stimulus. For the occluded condition, the measured noise level in response to the different stimuli was above the noise floor of the equipment and environmental noise within the sound-attenuating anechoic chamber. To ensure correct fitting, the first author (N.F.) fitted HPDs to the HATS.

#### 2.1.6. Data Analysis

Attenuation levels for pure tones were defined as the difference between the threshold of hearing levels without and with HPD. A one-sample *t*-test was used to compare the attenuation levels measured with the pCEP to those of 3M™-Combat-Arms™4.1 [26] and 3M™ E-A-R™ Classic™ [27]. Descriptive statistics were used to present data for objective attenuation level measured for the localization stimuli using the HATS.

### 2.2. Results

**Figure 2:**
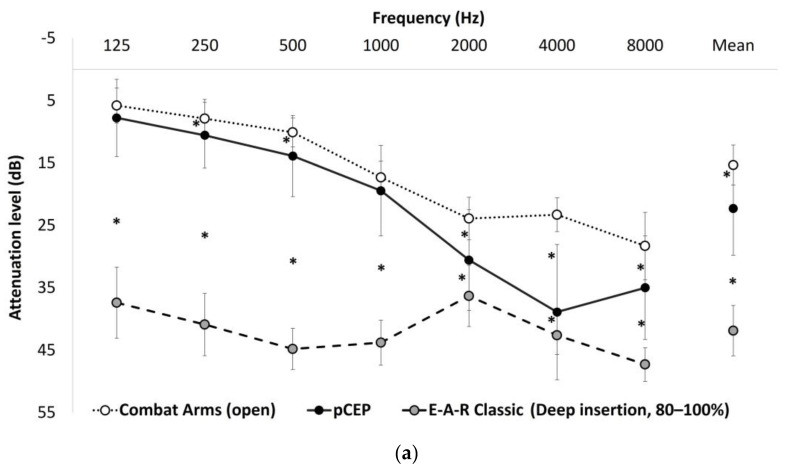

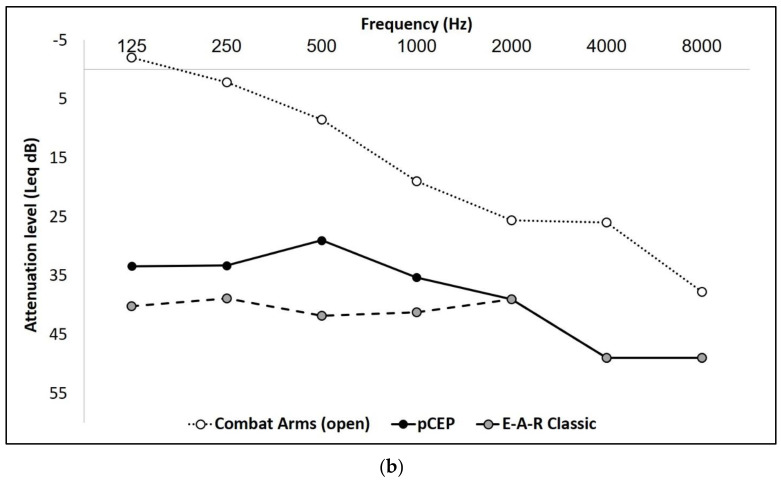
Attenuation levels of the three HPDs tested: (**a**) means and standard deviations for frequencies 125–8000 Hz, averaged across frequencies, as measured using the REAT method; (**b**) sound attenuation of white noise stimuli, displayed in the octave band spanning frequencies from 125–8000 Hz, as measured by a Head and Torso Simulator (HATS) with the 3M™-Combat-Arms™4.1 in open-mode, the 3M™ E-A-R™ Classic™ earplugs, and the pCEP. (**a**) Real Ear Attenuation (REAT) for pure tone frequencies 125-8000 Hz and averaged across frequencies. * *p* < 0.05. (**b**) Attenuation of white noise as measured by a Head and Torso Simulator (HATS).

Attenuation levels of the pCEP, 3M™ E-A-R™ Classic™, and 3M™-Combat-Arms™4.1 in open mode are presented in Figure 2a,b for the REAT and HATS methods, respectively. As can be seen in Figure 2a, a significant difference was found between the HPD attenuation levels of pure tones. Overall, the 3M™ E-A-R™ Classic™ demonstrated the largest attenuation level, followed by the pCEP, and then the 3M™-Combat-Arms™4.1 in open mode, which evidenced the smallest attenuation level. One-sample *t*-tests (corrected for multiple comparisons) showed that for all frequencies, the 3M™ E-A-R™ Classic™ presented significantly more attenuation than the pCEP. The 3M™-Combat-Arms™4.1 earplug in open mode presented significantly smaller attenuation than the pCEP in all frequencies, except for 125 Hz and 1000 Hz. Figure 2b shows that the largest attenuation of white noise was demonstrated by 3M™ E-A-R™-Classic™, the smallest attenuation was demonstrated by 3M™-Combat-Arms™4.1, and the pCEP demonstrated mid-range attenuation.

## 3. Experiment 2: Localization

### 3.1. Materials and Methods

#### 3.1.1. Participants

Experiment 2 enrolled 90 individuals, aged 20–35 years, who were screened for normal hearing (i.e., hearing thresholds ≤ 25 dB HL in frequencies of 500, 1000, 2000, 3000, 4000, 6000, and 8000 Hz). Exclusion criteria for Experiment 2 included a diagnosis of learning disability or attention deficit hyperactivity disorder, due to their association with a deficit in temporal processing [28,29]. Participants were divided into three groups of 30 participants each to measure the effect of the three different HPDs. The data from one group of 30 participants (3M™-Combat-Arms™4.1 group) was reported previously [30].

#### 3.1.2. Stimuli

The localization stimuli in the study included the impulse noise of an M16 assault rifle single gunshot and three consecutive gunshots (both recorded at a distance of 200 feet from the shooter); continuous pink noise; and a spoken word consisting of the Hebrew word “esh” (fire) spoken by a male speaker. The durations of the four stimuli were 202 ms for the single gunshot and an additional reverberating tail of 800 ms, 660 ms for the three gunshots with an additional reverberating tail of 660 ms, 212 ms for the pink noise, and 409 ms for the spoken word. All stimuli were presented at 65 dB SPL. The spectrum of each stimulus, as recorded using a GRAS 45CB HATS, was narrowest for the spoken word and widest for the pink noise (see Figure S2a). The spoken word also had the least accumulated energy $\left(E_{N}\right)$ over time, and the three gunshots had the most (see Figure S2b). All stimuli were presented at 65 dB SPL.

#### 3.1.3. Hearing Protection Devices (HPDs)

The same HPDs were used as detailed in Experiment 1.

#### 3.1.4. Apparatus

The localization experiment was carried out using the same apparatus described by Fostick and Fink (2021). A Dell™ Inspiron™ 13 5378 i5 laptop computer with a designated software written in C# with NET framework 4.5.2 was used to control the delivery of sound signals and log participant responses. The sounds were delivered through a Steinberg UR824 USB 2.0 Audio Interface™ into eight RCF Ayra 5^®^ active monitors. The experimental setup was calibrated by injecting each monitor separately with a 1000 Hz tone and measuring 100 dB SPL with the GRAS 45CB HATS™, the specifications of which were designed to comply with the ANSI/ASA S12.42 [31] standard. The HATS was situated in the center of a circle, 60 cm from each monitor, resembling the position of the human participants in this experiment. The calibration tone signal from each monitor was recorded with two 1/2″ microphones embedded in the HATS’s pinnae that were connected to a SINUS SAMURAI™ sound level meter conforming to IEC60651/IEC 60804/IEC 61672-1, IEC 651, and IEC 804 standards.

#### 3.1.5. Procedure

The procedure was the same as reported by [30] and is graphically depicted in Figure S3. The protocol for Experiment 2 was approved by the university ethical committee before the commencement of the research, and signed informed consent was obtained from all participants.

Each participant sat alone in a sound-attenuating anechoic chamber in the center of a circle of eight monitors positioned 60 cm from the participant and separated by 45°, starting from 22.5° and continuing through 337.5°. While the monitors were visible to the participants, their gaze was focused on a computer in tablet mode on their lap. The participants were asked to indicate the location of the source of each perceived sound on the computer screen’s display of a circle, the center of which contained an icon indicating the orientation of the participant. The screen displayed no indication of the monitors’ positions.

All stimuli were randomly delivered by the experimental software. Participants received a short training session to familiarize them with the experimental task. The training included 32 trials of one stimulus presentation from each monitor (4 stimuli type × 8 monitors), randomly intermixed by the experimental software. In the experiment, each stimulus was delivered 10 times from each monitor, resulting in 320 randomly presented trials (4 stimuli type × 8 monitors × 10 repetitions). After every 32 trials, the participants were offered a short break. The additional break was suggested between training and the beginning of the active experiment. Subsequently, half of the participants in each group were tested first with the HPD and then without the HPD, and the other half in the opposite order. Completing each condition (no HPD/with-HPD) lasted about 20 min, and the entire procedure (including screening and training) was almost 60 min. Participants received monetary compensation (USD 60) for their time.

#### 3.1.6. Data Analysis

Localization accuracy was calculated as the root mean square error (RMSE) of the angular difference between the response angle and the target monitor angle. Repeated measures ANOVAs were performed on mean RMSEs with condition (no-HPD, with-HPD), stimuli type (one gunshot, three gunshots, pink noise, and spoken word), and monitor angle (22.5°, 67.5°, 112.5°, 157.5°, 202.5°, 247.5°, 292.5°, 337.5°) as within-subjects variables, and HPD type (pCEP, 3M™-Combat-Arms™4.1, 3M™ E-A-R™ Classic™) as a between-subjects variable. For interaction and post hoc analyses, additional repeated measures ANOVAs, one-way ANOVAs, and LSD tests were used. Since HPDs significantly decrease localization accuracy, localization was analyzed separately for baseline (no-HPD) and with-HPD conditions.

### 3.2. Results

Table 1 presents descriptive and inferential statistics for the study variables. Overall, the mean RMSE of the pCEP was smaller than that of the 3M™ E-A-R™ Classic™ and similar to that of 3M™-Combat-Arms™4.1. The largest mean RMSE was found for the spoken word stimulus, followed by pink noise; the single gunshot; and, finally, the smallest for three gunshots. The largest mean RMSE was obtained when stimuli were delivered from the back (157.5° and 202.5°), and the smallest for the front and side angles (67.5°, 112.5°, 292.5°, and 337.5°). As expected, there was a larger mean RMSE for the with-HPD condition than for the no-HPD condition, roughly twice as large (40.85 and 21.86, respectively).

**Figure 3:**
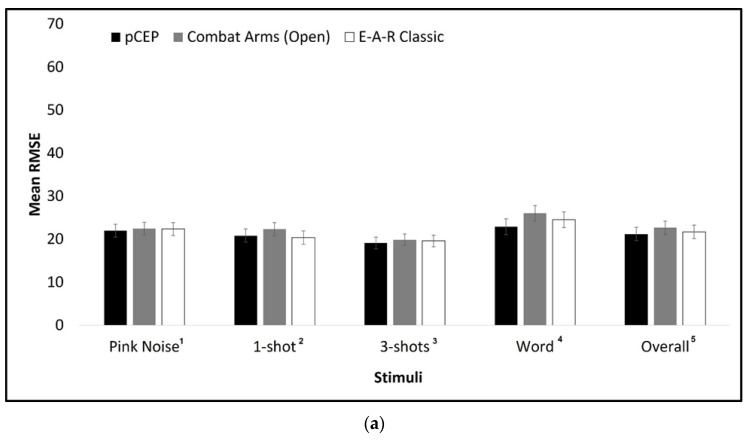

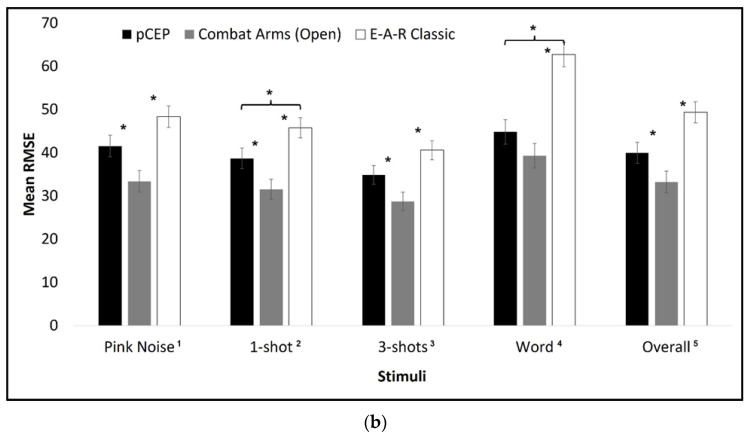
Mean RMSE and SE for each stimulus and overall mean RMSE across different stimuli for the various HPD groups for (**a**) no-HPD and (**b**) with-HPD conditions. (**a**) no-HPD (for the three subject groups divided according to HPD type). ^1^ *F*(2,89) = 0.029, *p* = 0.971; ^2^ *F*(2,89) = 0.437, *p* = 0.647; ^3^ *F*(2,89) = 0.088, *p* = 0.916; ^4^ *F*(2,89) = 0.712, *p* = 0.493; ^5^ *F*(2,89) = 0.272, *p* = 0.763. (**b**) with-HPD. * *p* < 0.05.

Significant interactions for HPD type were found with condition (*F*(2,87) = 13.304, *p* < 0.001, pη^2^ = 0.234), stimuli (*F*(6261) = 5.173, *p* < 0.001, pη^2^ = 0.106), and with condition and stimuli (*F*(6261) = 6.211, *p* < 0.001, pη^2^ = 0.125), but not with monitor angle (*F*(14,609) = 1.406, *p* = 0.145, pη^2^ = 0.031). Figure 3a,b illustrates the interactions of HPDs, conditions, and stimuli. As can be seen in Figure 3a, HPD groups did not differ in the baseline condition when no HPDs were used, but only in the with-HPD condition (Figure 3b). The general pattern of the difference between HPDs was similar across the various stimuli, with the largest mean RMSE for the 3M™ E-A-R™ Classic™ and the smallest for the 3M™-Combat-Arms™4.1, across all stimuli. The mean RMSE for the pCEP always fell between the mean RMSE of the other two HPDs: For pink noise and three gunshots, the pCEP RMSE was close to the 3M™ E-A-R™ Classic™, similar to the 3M™-Combat-Arms™4.1 for the spoken word, and different from both HPDs for the single gunshot.

## 4. Discussion

Soldiers’ need for acoustic communication while using HPDs is important. Yet, reducing or increasing sound intensity does not correlate with, or ensure, the sound clarity [3]. Undoubtedly, it is challenging to develop hearing protection from blasts and impulse noise while preserving auditory perception critical in military situations [17], yet among soldiers in military scenarios, there is a very high demand for an earplug that will protect their ears without compromising their ability to detect distant activity sounds. Such earplugs can attenuate 25 dB at high frequencies but may disrupt threat detection of a quiet enemy approach or understanding critical communications from fellow soldiers, especially if the background noise level is low [32].

The present study aimed to compare the proof-of-concept pCEP to the 3M™-Combat-Arms™4.1 earplugs in open-mode and the 3M™ E-A-R™ Classic™ earplugs in a noise attenuation measurement (Experiment 1) and a localization task (Experiment 2). In general, and as hypothesized, the performance of participants using the pCEP fell in between these two commercial earplugs in both noise attenuation and ability to localize sounds. The main findings showed that (1) localization abilities worsened when using HPDs; (2) the spoken word was localized less accurately than the other stimuli; (3) the mean RMSE was largest for stimuli emanating from rear monitors; and (4) localization abilities corresponded to HPD attenuation levels, with the largest attenuation level corresponding to the largest mean RMSE (both for the 3M™ E-A-R™ Classic™), lowest attenuation levels corresponding to the lowest mean RMSE (both for the 3M™-Combat-Arms™4.1 earplugs in open-mode), and the pCEP falling mid-range for both.

Achieving a larger mean RMSE when using HPDs than without HPDs is not surprising [33,34,35]. HPDs attenuate stimulus levels, markedly decreasing the amount of information delivered to the ear [36,37,38,39,40,41,42,43]. Although a recent meta-analysis showed that HPDs do not affect localization (and speech perception) despite sound attenuation [44], a reduction in localization performance was observed here for all three different HPDs. The decrease in localization ability with HPDs observed here and in other studies emphasizes the need for an HPD that can provide sufficient attenuation yet enable good user localization abilities [3]. The results from the 3M™ E-A-R™ Classic™ earplugs and the 3M™-Combat-Arms™4.1 earplugs demonstrate this point, with the 3M™ E-A-R™ Classic™ earplugs facilitating the largest attenuation level but also the poorest localization abilities as compared with the 3M™-Combat-Arms™4.1 earplugs that had the smallest attenuation with the best localization.

The proof-of-concept pCEP seems to answer the challenge of an HPD that can present high attenuation levels and yet enable localization ability. It possesses the attenuation advantage of the 3M™ E-A-R™ Classic™ earplugs by being made from the same material (polyurethane) that expands in the ear canal, clings to its walls, and blocks the canal. It also demonstrates less localization errors compared to the 3M™ E-A-R™ Classic™ earplug. This may be due to the narrow tube within the earplug that was suggested by Casali and Berger [21] to enable the transition of soft sounds and low-frequency components of sound. An earplug with a narrow tube may allow better localization compared to earplugs lacking a narrow tube, such as the 3M™ E-A-R™ Classic™ earplug. In addition, the concave-bowl-like rigid structure that faces the lateral side and the narrow diameter of the pCEP tube (narrower than that of the 3M™-Combat-Arms™4.1) enhances attenuation further with little cost to the localization RMSE. Overall, localization abilities using the pCEP were better than those of the 3M™ E-A-R™ Classic™ earplugs and worse than those of the 3M™-Combat-Arms™4.1 earplugs, as would be expected by their relative attenuation level. However, the pCEP was significantly better than the 3M™ E-A-R™ Classic™ earplugs in localizing the spoken word and gunshot sounds, despite a similar attenuation level (see Figure 2b). The pCEP also showed no difference in localizing the spoken word compared to the 3M™-Combat-Arms™4.1 earplugs, although evidencing a larger attenuation; this may be attributed to its narrower tube that enables larger attenuation with little effect on the localization of the spoken word.

Two additional interesting findings were observed in the current study. The first was a difference in localization ability between the various stimuli. As reported previously, the stimulus spectrum affects the ability to localize it [30,38,45,46,47,48,49,50,51,52,53]; indeed, in the present study, the spoken word (the word “esh” (fire) in Hebrew) had lower sound levels in the low and high frequencies in comparison to the spectra of both of the gunshot stimuli and the pink noise. Less spectral information causes larger localization errors, with and without HPDs, regardless of the HPD type. This finding corresponds to previous studies that tested speech stimuli in comparison to other stimuli and found that speech was localized poorer than wider-spectra stimuli [30,45,46,48].

The findings also showed that the three-gunshot stimulus was localized better than a single gunshot, although both have a similar spectrum. This may be explained by the longer duration of the three-gunshot noise relative to the single gunshot: sounds with longer duration not only provide more accumulated energy for the listener to grasp and process but also provide the listener with sufficient time for head movement toward the direction of the sound. Both of these factors improve localization [40,54,55].

Another interesting finding is related to differences in localization between monitor angles. The most notable finding, which can be also observed in other localization studies, was poor localization of sound stimuli emanating from the rear monitors (157.5° and 202.5°) [19,30,36,37,38,40,54]. A probable explanation for this is that stimulus delivered from the left and right of the head has large interaural time and level differences than from the back of the head [54]. Therefore, the localization of stimulus delivered from the back of the head relies on spectral cues that are weaker than those used for localizing stimuli delivered from the left and right [40].

One limitation of the current study is that comparisons between HPDs were made between groups of different participants, rather than in a within-subjects design. This was done to avoid too lengthy an experiment, which already included comparisons between four stimuli and two conditions. The random assignment to HPD groups and the absence of group differences at baseline may have compensated for this limitation, supporting the conclusion that group differences are due to differences between HPDs. Another limitation consists of the relatively short distance between the monitors and the participant (60 cm)—compared to the accustomed distance suggested for near-field (<1 m from the sound source)—that may have affected localization. This distance was used in the current study based on the limited dimensions of the sound-attenuating anechoic chamber used. The rule of thumb for calculating the far-field distance from the sound source is 1 wavelength of the sound signal or three times the largest dimension of the sound source. At 60 cm away from the monitor, according to the 1-wavelength rule of sound, any sound frequencies higher than 1700 Hz of the sound signal would be in the far-field while lower frequencies would be in the near-field. According to the “three times the largest dimension of the sound source” rule of thumb, the far-field of sounds delivered from the RCF Ayra 5^®^ active monitor (1” tweeter; 5” woofer) was 15 inches or 38 cm, thus placing our participants in the far-field at 60 cm. Previous studies indicate that interaural time differences remain constant when moving from far- to near-field, but interaural level differences increase [56,57]. Therefore, because differences in sound localization might be smaller in the far-field, future investigations are recommended to position monitors at a greater distance if possible.

A further limitation of the study relates to the method of measuring hearing thresholds to calculate the attenuation levels of the various HPDs. Hearing thresholds were obtained with pure tones rather than with narrow band or warble tones, which are more accustomed and instructed according to ANSI/ASA S12.6 [57]. However, Norrix and Anderson [58] reported several studies showing thresholds obtained using narrow-band noise and/or warbled noise comparable to those obtained with pure tones in adults. Notwithstanding, in the future, a finalized version of the pCEP will be evaluated using narrow-band noise according to ANSI/ASA S12.42. [35] and ANSI/ASA S12.6. [57], as well as that compared to other HPDs.

A final limitation to mention is that the proof-of-concept pCEP is an open-mode HPD that enables the passage of sound or noise, although attenuated to some extent. A prototype currently under evaluation incorporates an embedded cap on the outward-facing end of the pCEP. When the cap is maneuvered into the closed mode, it blocks the entrance to the narrow tube and adds further attenuation. The ability of the next prototype to switch between closed and open modes will enable soldiers to use the HPD in both mounted and dismounted scenarios, respectively.

## 5. Conclusions

This study demonstrated that high efficacy noise attenuation HPDs, such as the 3M™ E-A-R™ Classic™, have a downside of increasing localization errors. On the other hand, earplugs that improve localization accuracy, such as 3M™-Combat-Arms™4.1 in open mode, do so at the cost of poor noise attenuation. The present study demonstrated that a proof-of-concept earplug, the pCEP, combines, to some extent, the advantages of sound localization and noise attenuation; it provides improved sound localization relative to 3M™ E-A-R™-Classic™ and a higher level of attenuation relative to 3M™-Combat-Arms™4.1. These findings suggest that pCEP earplugs may be an HPD to consider for use in military settings in which sound localization, as well as noise attenuation, are critical for mission success and service members’ protection. Further research, consisting of additional factor component examinations and a large-scale user experiment, will be performed in the process of evaluating the pCEP. This includes evaluation by professional soldiers in mounted- and dismounted-soldier scenarios, who will be asked to evaluate pCEP performance in low- and high-intensity military noise scenarios, as well as during tasks of sound localization and speech recognition and intelligibility.

## Figures and Tables

**Table 1:** RMSE mean (SE), main effects for repeated measures analysis, and post hoc LSD test for the study variables.

	Mean (SE)	Main Effect		Post Hoc
		F	pη^2^	LSD Test
*Condition*		197.86 ***	0.7	
HPD	40.85 (1.31)			
No-HPD	21.86 (0.82)			
*HPD type*		6.65 **	0.13	
pCEP	30.58 (1.49)			pCEP vs. E-A-R™ Classic™: 4.95 * pCEP vs. 3M™-Combat-Arms™4.1: 2.64 E-A-R™ Classic vs. 3M™-Combat-Arms™4.1: 7.59 **
E-A-R™ Classic™	35.54 (1.49)			
3M™-Combat-Arms™4.1	27.95 (1.49)			
*Stimuli*		50.27 ***	0.64	
Word	36.71 (1.11)			word vs. pink noise: 0.04 *** word vs. M16 1-shot: 6.79 *** word vs. M16 3-shots: 9.58 *** pink noise vs. M16 1-shot: 1.75 ** pink noise vs. M16 3-shots: 4.54 *** M16 1-shot vs. M16 3-shots: 2.79 ***
Pink noise	31.68 (0.93)			
Single gunshot	29.92 (0.91)			
Three gunshots	27.13 (0.76)			
*Monitor angle*		23.59 ***	0.67	
22.5	30.04 (1.83)			see Table S1
67.5	25.5 (0.84)			
112.5	26.92 (1.04)			
157.5	46.39 (1.92)			
202.5	39.32 (1.76)			
247.5	29.13 (1.08)			
292.5	25.27 (1.00)			
337.5	28.33 (1.64)			

* *p* < 0.05; ** *p* < 0.01; *** *p* < 0.001.

## Data Availability

The data presented in this study are available on request from the corresponding author. The data are not publicly available due to the ongoing analysis of the data.

## Supplementary Materials

The following supporting information can be downloaded at https://www.mdpi.com/article/10.3390/s23177410/s1, Figure S1: The pCEP in conjunction with a commercial “listen only” acoustic tube. Figure S2: Description of single and triple M16 gunshots at a distance of 200 feet, pink noise, and a spoken word (Hebrew word “esh” (fire)), showing (a) spectrum and (b) cumulative energy). Figure S3: Experimental setting. Table S1: Results of post hoc LSD analyses between all monitors angles.
