# Supplementary material for: Perforated Concave Earplug (pCEP): A Proof-of-Concept Earplug to Improve Sound Localization without Compromising Noise Attenuation
Source: Sensors (Basel). 2023 Aug 25;23(17):7410. doi: 10.3390/s23177410 (PMC10490414; doi:10.3390/s23177410)
Supplement: Supplementary file 1 [file sensors-23-07410-s001.zip › sensors-2519672-supplementary.pdf]

Supplementary Materials

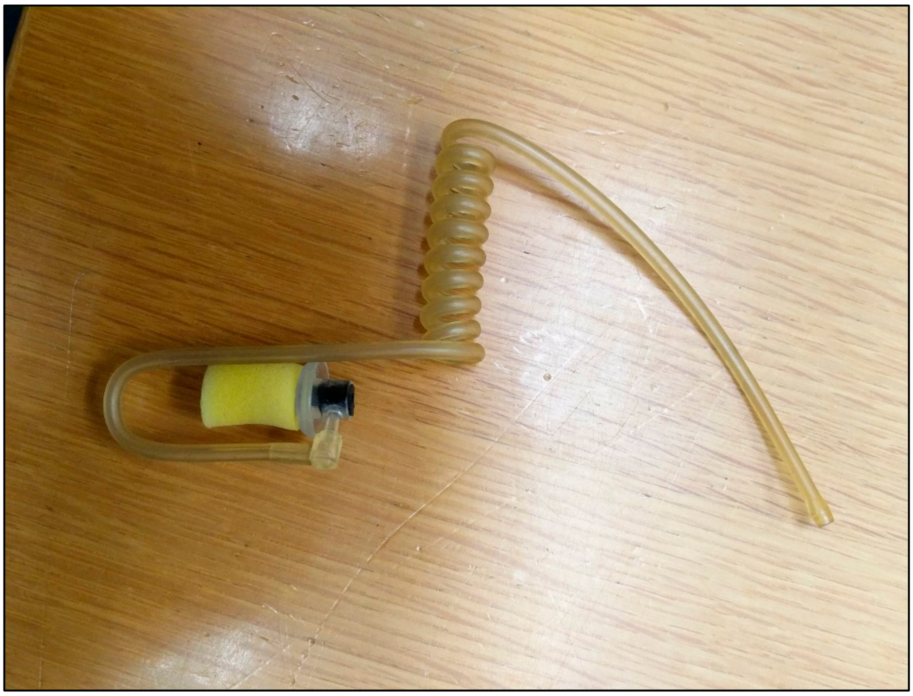

Figure S1: The pCEP in conjunction with a commercial "listen only" acoustic tube. The pCEP may be further used in conjunction with a commercial "listen only" acoustic tube, an accessory supplied by tactical radio manufacturers for the dismounted soldier, which is displayed connected to the pCEP. This may allow for voiced radio-transmission to pass along the narrow tube and to reach the user's eardrum while being protected from surrounding noise. No results of speech intelligibility and localization are presented.

a. Spectrum

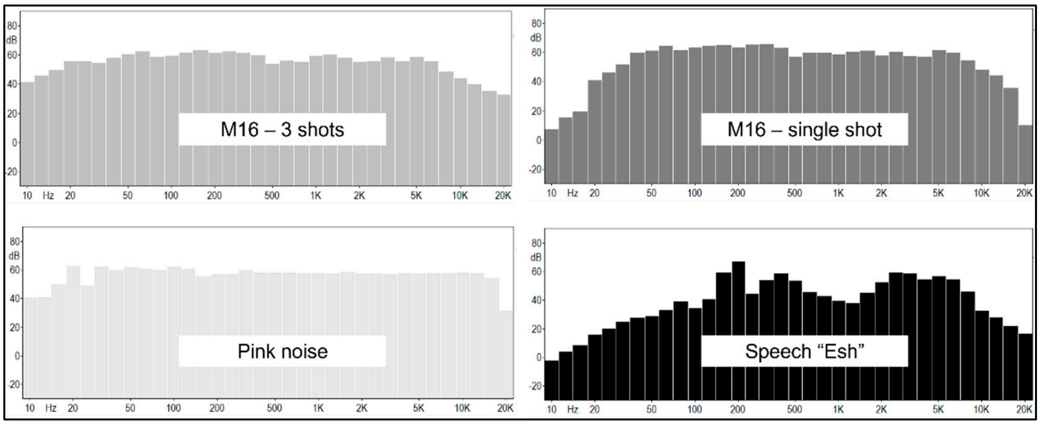

b. Cumulative energy

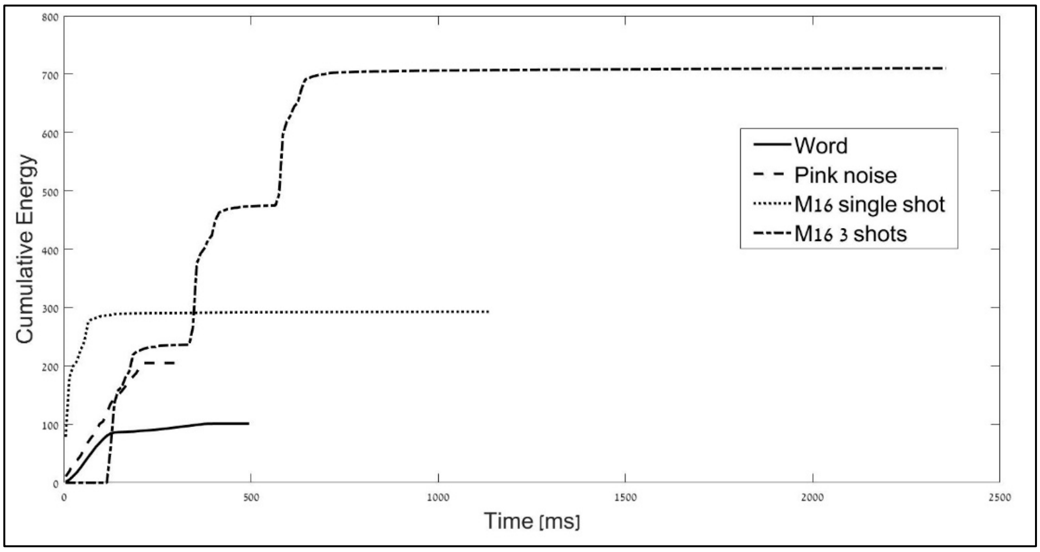

Figure S2: Description of single and triple M16 gunshots at a distance of 200 feet, pink noise, and a spoken word (Hebrew word /esh/ (fire)), showing: (a) spectrum and (b) cumulative energy).

The Cumulative Energy unit corresponds to the (-1) – (+1) full-scale amplitude range of the WAV files of the stimuli. The energy of a discrete-time signal over a finite interval $-N \le n \le N$ is given by:

$$E_N = \sum_{-N}^{N} |x(n)|^2$$

where N=220.5. The energy was calculated using a 10 ms sliding window (fs = 44,100Hz, window size = fs/100 = 441). Cumulative energy was obtained by summing the energies of all sliding windows of each stimulus.

a. Actual setting b. Setting presented to the participants

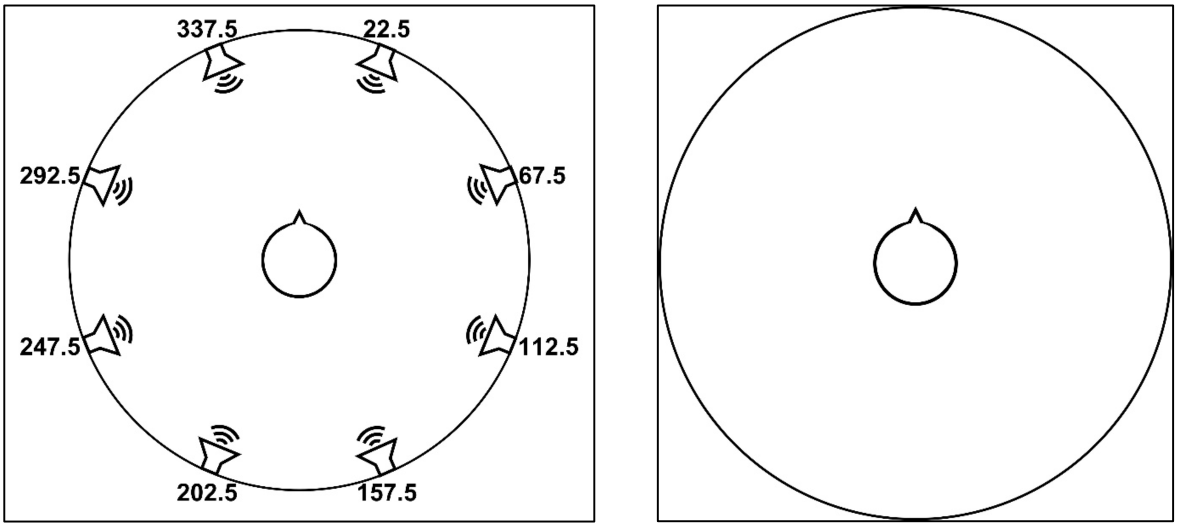

Figure S3: Experimental setting. The symbol in the center represents participant orientation. (a) The actual setting. The participant sat in the center of a circle of eight monitors separated by 45°, starting from 22.5° through 337.5°. (b) The image was presented to participants via computer screen, with no indication of the monitors' positions. Participants were asked to indicate on the circle the location of the perceived sound source.

Table S1. Results of post-hoc LSD analyses between all monitors angles (Significant comparisons are highlighted in grey shading).

		Back	Left		Front		Right		Back
		**202.5°**	**247.5°**	**292.5°**	**337.5°**	**22.5°**	**67.5°**	**112.5°**	**157.5°**
**Back**	**202.5°**		10.19	14.05	10.98	-9.27	-13.82	-12.40	7.07
**Left**	**247.5°**			3.86	.80	.91	-3.63	-2.21	17.26
	**292.5°**				-3.07	4.77	.23	1.65	21.12
**Front**	**337.5°**					1.71	-2.84	-1.42	18.06
	**22.5°**						4.55	3.13	-16.35
**Right**	**67.5°**							-1.42	-20.89
	**112.5°**								-19.47
